# Adherence to oral therapies among patients with renal cell carcinoma: Post hoc analysis of the ECOG‐ACRIN E2805 trial

**DOI:** 10.1002/cam4.4140

**Published:** 2021-08-18

**Authors:** Caitlin C. Murphy, Hannah M. Fullington, David E. Gerber, Isaac Alex Bowman, Maneka Puligandla, Janice P. Dutcher, Robert S. DiPaola, Naomi B. Haas

**Affiliations:** ^1^ School of Public Health University of Texas Health Science Center at Houston (UTHealth) Houston TX USA; ^2^ Department of Population and Data Sciences University of Texas Southwestern Medical Center Dallas TX USA; ^3^ Department of Internal Medicine University of Texas Southwestern Medical Center Dallas TX USA; ^4^ Banner MD Anderson Cancer Center Gilbert AZ USA; ^5^ Department of Data Sciences Dana‐Farber Cancer Institute Boston MA USA; ^6^ Cancer Research Foundation of New York Chappaqua NY USA; ^7^ College of Medicine University of Kentucky Lexington KY USA; ^8^ Division of Hematology‐Oncology Perelman School of Medicine University of Pennsylvania Philadelphia PA USA

**Keywords:** adherence, clinical trial, renal cell carcinoma

## Abstract

**Background:**

As use of oral cancer therapies increases, patient adherence has become critical when evaluating the effectiveness of therapy. In a phase III trial for renal cell carcinoma, we: (a) characterized adherence to sorafenib, sunitinib, and/or placebo and (b) identified factors associated with non‐adherence.

**Methods:**

ECOG‐ACRIN E2805 was a double‐blind, placebo‐controlled, randomized trial comparing adjuvant sorafenib or sunitinib in patients with resected primary renal cell carcinoma at high risk for recurrence. We used patient‐completed pill diaries to measure adherence as the number of pills taken divided by the number of pills prescribed. Log‐binomial regression was used to identify correlates of non‐adherence (<80% of prescribed pills reported as taken).

**Results:**

Mean adherence was 90.7% among those assigned to sunitinib (n = 613) and 84.8% among those assigned to sorafenib (n = 616). Among those assigned to placebo, mean adherence was 94.9% and 92.4% to sunitinib and sorafenib placebo, respectively. Non‐adherence was associated with race/ethnicity (non‐Hispanic Black: prevalence ratio [PR] 2.22, 95% CI 1.63, 3.01; Hispanic: PR 1.54, 95% CI 1.05, 2.26), high volume enrollment (≥10 patients: PR 1.30, 95% CI 1.03, 1.64), treatment group (sunitinib: PR 2.24, 95% CI 1.66, 3.02; sorafenib: PR 2.37, 95% CI 1.74, 3.22), and skin rash (PR 1.36, 95% CI 1.03, 1.80).

**Conclusion:**

Among patients participating in a randomized clinical trial, adherence to oral cancer therapies was lower compared to placebo. Adherence was also worse in racial/ethnic minorities, those experiencing toxicities, and high volume enrolling sites. Our findings highlight several challenges to address in clinical practice as use of oral therapies continues to increase.

**Clinical trial registration number:**

This trial is registered with ClinicalTrials.gov, number NCT00326898.

## Graphical abstract

In a randomized clinical trial for renal cell carcinoma, adherence to oral therapy was lower (85%) than adherence to placebo (95%). Adherence was also worse among racial/ethnic minorities, patients experiencing certain toxicities, and high volume enrolling sites, highlighting several challenges to address in clinical practice as oral therapies become increasingly common.

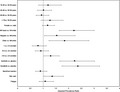

## 1 BACKGROUND

The landscape of cancer treatment now includes many oral regimens, raising new concerns for oncology teams’ supervision, and delivery of high‐quality cancer care.^1^, ^2^, ^3^, ^4^ Oral cancer therapies comprise nearly one third of all anticancer agents, and many cancer therapies in the pipeline are being developed exclusively as oral regimens. Chemotherapy administration has shifted from a safe, controlled, in‐clinic process monitored by oncology teams to patients’ homes, where providers have no direct supervisory role. As use of oral cancer therapies increases, many traditional responsibilities of providers, including adhering to dosing decisions and identifying toxicities, have moved more directly to patients and caregivers.^1^ Patients are asked to adherence to complex dosing regimens that may change frequently due to toxicities. Patients may also be uncertain about interactions with other prescription medications or supplements^5^, ^6^ and how to manage late or missed doses.

Adherence has become critically important when interpreting treatment outcomes in the context of clinical trials. Yet, adherence is rarely reported in clinical trials evaluating the efficacy of oral therapies. Lack of adherence data may lead to inaccurate conclusions about dosage requirements, therapeutic effectiveness, or toxicity of a drug regimen. In a phase III trial for non‐metastatic renal cell carcinoma, we: (a) characterized patient adherence to sorafenib, sunitinib, and/or placebo and (b) identified patient‐ and site‐level factors associated with adherence.

## 2 METHODS

### 2.1 Study population

The ECOG‐ACRIN Cancer Research Group (ECOG‐ACRIN) led a double‐blind, placebo‐controlled, randomized phase III trial comparing disease‐free survival (DFS) with adjuvant sorafenib or sunitinib in patients with resected primary renal cell carcinoma at high risk for recurrence (E2805).^7^ Eligible patients had histologically proven, completely resected high‐risk clear cell or non‐clear cell renal cell carcinoma and were within 12 weeks of removal of the primary tumor. Patients were randomly assigned (1:1:1) to receive 54 weeks of sunitinib 50 mg per day orally for the first 4 weeks of each 6‐week cycle; sorafenib 400 mg twice per day orally throughout each 6‐week cycle; or placebo. Placebo could be sunitinib placebo for the first 4 weeks of each 6‐week cycle or sorafenib placebo throughout. Sunitinib or sunitinib placebo was administered as four 12.5 mg pills; sorafenib or sorafenib placebo was administered as two 200 mg pills. Therefore, regardless of treatment group, all patients were on the same schedule with the same number of pills.

The primary outcome analysis showed no significant differences in DFS across treatment groups. Median DFS was 5.8 years for sunitinib (HR 1.02, 97.5% CI 0.85, 1.23), 6.1 years for sorafenib (HR 0.97, 97.5% CI 0.80, 1.17), and 6.6 years for placebo.

### 2.2 Measures

Patients used a pill diary to record the number of pills taken each day and time taken. Pill diaries and bottles were returned every 6 weeks during visits at the end of each treatment cycle. We used these data to measure adherence as the number of pills taken divided by the number of pills prescribed. If doses were modified (planned or unplanned), we adjusted the number of pills prescribed accordingly. For example, a patient randomly assigned to sorafenib reporting 265 pills taken (out of 378 pills prescribed over nine 6‐week treatment cycles requiring 42 consecutive daily doses in each cycle, no dose modifications) was 70% adherent. We calculated adherence across all treatment cycles up until the date of disease progression, treatment discontinuation, or death.

### 2.3 Statistical analysis

We used log‐binomial regression to identify correlates of non‐adherence. Patients were categorized as non‐adherent if <80% of prescribed pills were reported as taken.^8^, ^9^, ^10^, ^11^ Potential correlates included: age, sex, race/ethnicity, treatment group, enrollment site type (academic, cooperative group oncology program, community clinical oncology program, and other), enrollment site volume (1, 2–5, 6–9, ≥10 patients), ECOG performance status, and select grade 3 or 4 adverse events (neuropathy, stomatitis, GI symptoms, hand–foot reaction, skin rash, joint pain, and fatigue). To build a multivariable model, we included age and sex a priori and selected variables significantly associated with non‐adherence in univariate analysis (*p* < 0.25). We report unadjusted and adjusted prevalence ratios (PR) and 95% confidence intervals.

In exploratory analyses, we used Cox proportional hazards regression to estimate the association between non‐adherence and overall survival (OS) and DFS. OS was calculated from randomization until the last known date of follow‐up or date of death, and DFS was calculated from randomization until date of disease progression, last known date of follow‐up, or date of death. To illustrate findings, we plotted cumulative incidence curves using Kaplan–Meier method and compared survival distributions by adherence (+/‐ 80%) using a log‐rank test.

Analyses were conducted using SAS version 9.4 (SAS Institute). This study was approved by the Institutional Review Board at the University of Texas Southwestern Medical Center (#052018‐006).

## 3 RESULTS

A total of 1,943 patients were randomly assigned to sunitinib (n = 647, 33.3%), sorafenib (n = 649, 33.4%), and placebo (n = 647, 33.3%). Of these, 1,858 (95.6%) patients completed pill diaries and were included in our analysis. Patient characteristics are shown in Table 1. Most patients were male (67.3%), non‐Hispanic White (87.0%), and enrolled at an academic center (43.7%). In the first treatment cycle, patients assigned to sunitinib and sorafenib were prescribed a median of 112 and 104 pills (non‐placebo), respectively.

**TABLE 1:** Characteristics of 1,858 patients randomized to sunitinib, sorafenib, or placebo, ECOG‐ACRIN E2805

	n	%
Age (years)		
18–39	118	6.4
40–49	384	20.7
50–59	660	35.5
60–69	497	26.8
≥70	199	10.7
Sex		
Male	1250	67.3
Female	608	32.7
Race/ethnicity		
Non‐Hispanic White	1617	87.0
Non‐Hispanic Black	78	4.2
Hispanic	100	5.4
Other	63	3.4
Enrolling site type		
Academic	812	43.7
CGOP	469	25.2
CCOP	564	30.4
Other	13	0.7
Enrolling site volume		
1	257	13.8
2–5	714	38.4
6–9	401	21.6
≥10	486	26.2
Performance status^a^		
0	1474	79.9
≥1	371	20.1
Missing	13	
Treatment group		
Sunitinib	613	33.0
Sorafenib	616	33.2
Placebo	629	33.9
Grade 3 or 4 adverse events		
Neuropathy	50	2.7
Stomatitis	91	4.9
GI symptoms^b^	309	16.6
Hand–foot reaction	359	19.3
Skin rash^c^	196	10.6
Joint pain	98	5.3
Fatigue	309	16.6

Abbreviations: CCOP, community clinical oncology program; CGOP, cooperative group oncology program.

^a^ Performance status at first study visit.

^b^ GI symptoms include nausea, vomiting, diarrhea, and constipation.

^c^ Skin rash also includes desquamation and acne.

Mean adherence to the study drug was 90.7% among those assigned to sunitinib (n = 613) and 84.8% among those assigned to sorafenib (n = 616) (Table 2). Among those assigned to placebo, mean adherence was 94.9% and 92.4% to sunitinib and sorafenib placebo, respectively.

**TABLE 2:** Mean adherence (proportion of pills prescribed reported as taken) by treatment group, ECOG‐ACRIN E2805 (n = 1,858)

	Study drug	Sunitinib placebo	Sorafenib placebo	All prescribed drugs
Treatment group				
Sunitinib (n = 613)	90.7 (95% CI 89.3, 92.1)	—	86.8 (95% CI 85.2, 88.5)	87.8 (95% CI 86.3, 89.2)
Sorafenib (n = 616)	84.8 (95% CI 82.8, 86.8)	88.0 (95% CI 86.2, 89.8)	—	85.4 (95% CI 83.6, 87.2)
Placebo (n = 629)	—	94.4 (95% CI 93.9, 95.9)	92.4 (95% CI 91.4, 93.5)	93.2 (95% CI 92.2, 94.2)

Note

All patients took four 12.5 mg pills of sunitinib or sunitinib placebo per day for the first 28 days of every 6‐week treatment cycle and two 200 mg pills of sorafenib or sorafenib placebo twice per day throughout.

**FIGURE 1:**
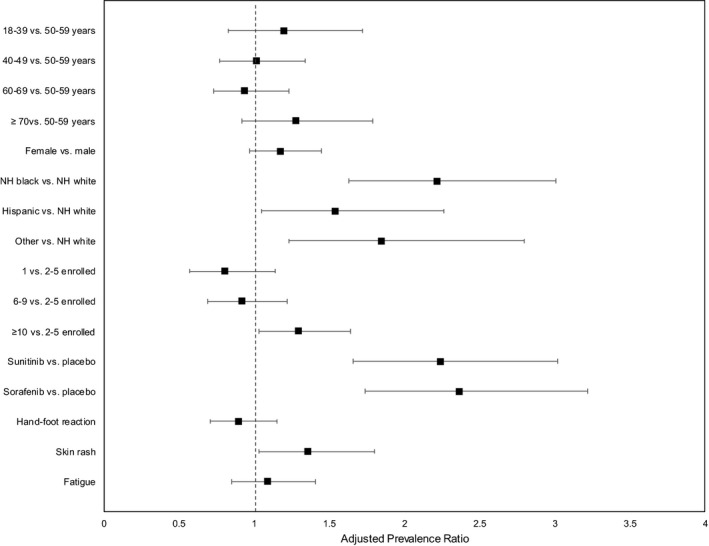
Forest plot of adjusted prevalence ratios, factors associated with non‐adherence (<80% of pills prescribed reported as taken) to study drug, ECOG‐ACRIN E2805 (n = 1,858)

In the adjusted log‐binomial model (Table 3, Figure 1), non‐adherence was statistically significantly associated with race/ethnicity (non‐Hispanic black: PR 2.22, 95% CI 1.63, 3.01; Hispanic: PR 1.54, 95% CI 1.05, 2.26; other: PR 1.85, 95% CI 1.23, 2.80), enrolling site volume (≥10 patients: PR 1.30, 95% CI 1.03, 1.64), treatment group (sunitinib: PR 2.24, 95% CI 1.66, 3.02; sorafenib: PR 2.37, 95% CI 1.74, 3.22), and skin rash (PR 1.36, 95% CI 1.03, 1.80).

**TABLE 3:** Factors associated with non‐adherence (<80% of pills prescribed reported as taken) to study drug, ECOG‐ACRIN E2805 (n = 1,858)

	Unadjusted		Adjusted	
	PR	95% CI	PR	95% CI
Age (years)				
18–39	1.33	0.91, 1.95	1.20	0.83, 1.72
40–49	0.99	0.75, 1.32	1.02	0.77, 1.34
50–59	1.00		1.00	
60–69	0.89	0.68, 1.17	0.94	0.73, 1.23
≥70	1.16	0.83, 1.61	1.28	0.92, 1.79
Sex				
Male	1.00		1.00	
Female	1.25	1.02, 1.54	1.18	0.97, 1.45
Race/ethnicity				
Non‐Hispanic White	1.00		1.00	
Non‐Hispanic Black	2.30	1.66, 3.19	2.22	1.63, 3.01
Hispanic	1.46	0.99, 2.16	1.54	1.05, 2.26
Other	1.80	1.18, 2.74	1.85	1.23, 2.80
Enrolling site type				
Academic	1.00			
CGOP	0.87	0.68, 1.12		
CCOP	0.73	0.57, 0.95		
Other	2.07	1.02, 4.18		
Enrolling site volume				
1	0.83	0.56, 1.18	0.81	0.57, 1.14
2–5	1.00		1.00	
6–9	0.88	0.66, 1.18	0.92	0.69, 1.22
≥10	1.24	0.98, 1.58	1.30	1.03, 1.64
Performance status^a^				
0	1.00			
≥1	1.11	0.87, 1.42		
Treatment group				
Sunitinib	2.20	1.63, 2.96	2.24	1.66, 3.02
Sorafenib	2.43	1.81, 3.25	2.37	1.74, 3.22
Placebo	1.00		1.00	
Adverse events				
Neuropathy	0.84	0.42, 1.68		
Stomatitis	1.27	0.84, 1.92		
GI symptoms^b^	1.04	0.79, 1.36		
Hand–foot reaction	1.29	1.02, 1.64	0.90	0.71, 1.15
Skin rash^c^	1.60	1.22, 2.09	1.36	1.03, 1.80
Joint pain	1.31	0.88, 1.94		
Fatigue	1.21	0.94, 1.56	1.09	0.85, 1.41

Abbreviations: CCOP, community clinical oncology program; CGOP, cooperative group oncology program.

^a^ Performance status at first study visit.

^b^ GI symptoms include nausea, vomiting, diarrhea, and constipation.

^c^ Skin rash also includes desquamation and acne.

**FIGURE 2:**
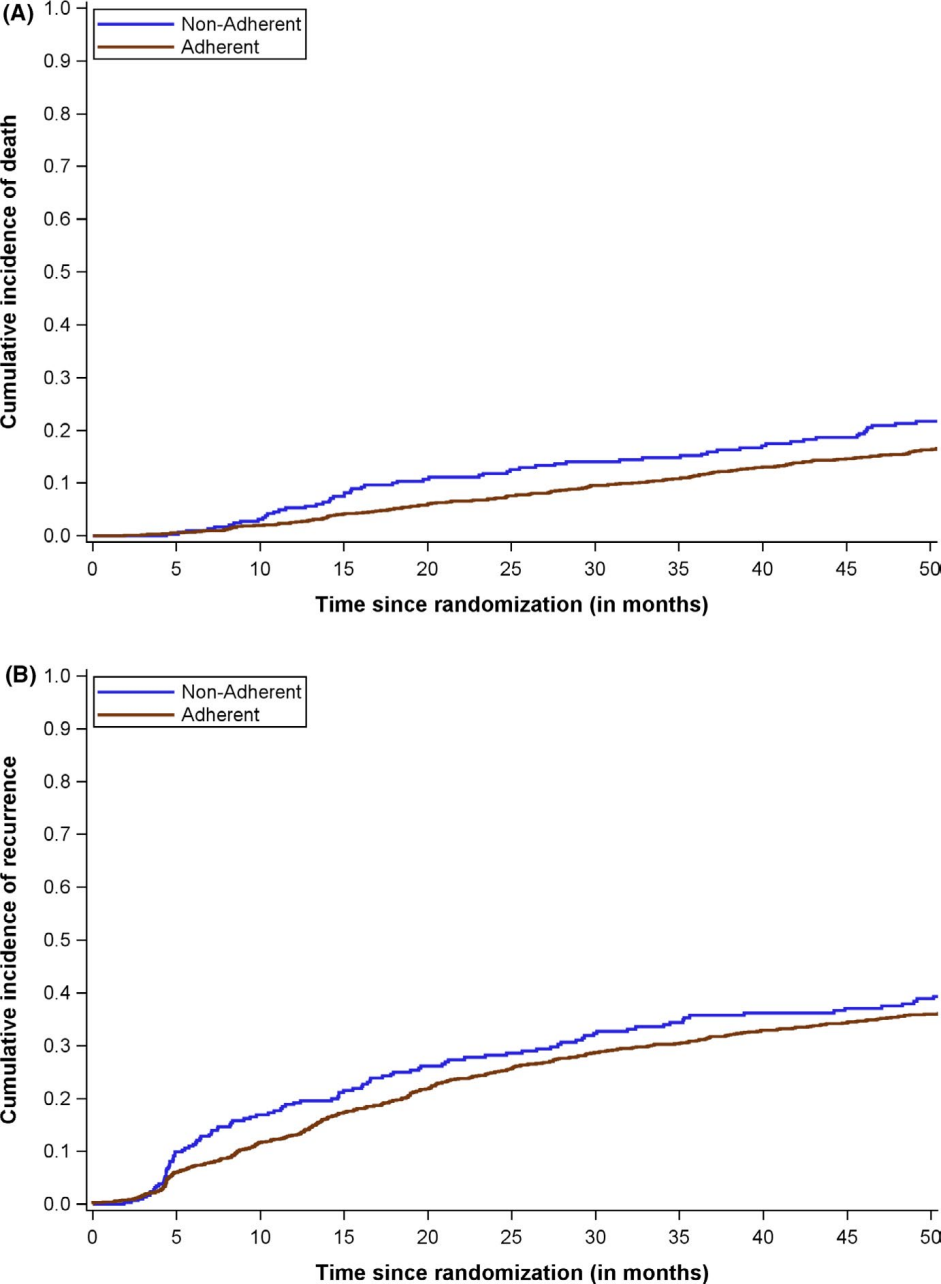
Cumulative incidence of death (A) and recurrence (B) by adherence to study drug, ECOG‐ACRIN E2805 (n = 1,858)

Non‐adherence was not statistically significantly associated with OS (HR 0.90, 95% CI 0.70, 1.14) or DFS (HR 0.88, 95% CI 0.72, 1.07), after adjusting for age, race/ethnicity, sex, and treatment group. Similarly, there was no difference in cumulative incidence of death or recurrence between adherent and non‐adherent patients (Figure 2).

## 4 DISCUSSION

Patient adherence to oral cancer therapies is a well‐recognized challenge of care,^4^ and poor adherence may undermine treatment efficacy.^12^, ^13^, ^14^, ^15^, ^16^ Across clinical trials, collecting pill counts and diaries is a time‐ and resource‐intensive effort, but this information is almost never reported. In a phase III trial for non‐metastatic renal cell carcinoma, we found generally good (>80%) adherence to oral therapies, although adherence to sunitinib and sorafenib was notably lower compared to placebo. Adherence also varied by patient‐ and system‐level factors––worse among racial/ethnic minorities, patients experiencing certain toxicities, and high volume enrolling sites.

Racial/ethnic minorities were less likely to adhere to oral therapies compared to non‐Hispanic Whites, even after adjusting for toxicities known to differ across groups.^17^, ^18^, ^19^ Others have similarly noted barriers to oral therapy adherence differ by race/ethnicity, including altered risk perceptions, misbeliefs about treatment efficacy, poor awareness of benefits, and fragmented communication with providers.^20^ The shifting treatment paradigm away from parenteral chemotherapy may negatively impact minority patients because of struggles to access and adhere to oral regimens. These patients face barriers to care: competing social and economic demands,^21^ adhering to complex dosing regimens, and identifying and reporting toxicities. Many are Spanish speakers or need help reading English language^22^ drug labels and dosing instructions. In clinical practice (vs. trials), high costs of oral regimens present an additional barrier to timely receipt of and adherence to therapy.^23^, ^24^, ^25^ Little is known about how patients and providers address and overcome these challenge, and the impact of oral therapies on cancer health disparities deserves urgent study.

Patients experiencing grade 3 or 4 toxicities, including skin rash and hand–foot reaction, had lower adherence to oral therapies. Other toxicities that we expected to be associated with adherence (e.g., fatigue, neuropathy) had little or no impact. Across cancer types, toxicities of oral therapy may act to promote adherence (because patients perceive an effect) or discourage adherence (to avoid symptoms).^26^ For example, in a qualitative study of patients prescribed oral cancer therapies,^27^ some patients were reluctant to report toxicities to their providers because they feared that dose reductions would compromise effectiveness of therapy. Others described delaying or forgoing therapy because of symptoms rather than reporting toxicities as they occurred. Toxicities may also change dosing regimens, perhaps introducing an additional challenge of monitoring patient adherence. Indeed, shortly after E2805 began, the starting doses of both sorafenib and sunitinib were amended to address toxicity issues, and revised dosing still resulted in high toxicity.^7^ Tools to facilitate communication between patients and providers (e.g., web‐based monitoring^28^) may allow patients receiving oral therapies to quickly report toxicities and receive tailored feedback regarding symptom management or dose modifications.

Despite our initial hypothesis, adherence was worse in high volume enrollment (≥ 10 patients) sites compared to sites enrolling only one or two patients. We expected high volume sites to have higher adherence, possibly reflecting more efficient and experienced processes related to delivering care to and monitoring patients on clinical trials. Instead, these sites may have relatively fewer resources and ancillary support. Research staff may have less time to devote to reviewing pill diaries, assessing barriers to adherence, and educating patients. Most of the medication adherence literature (for cancer and other chronic conditions) focuses on patient‐level factors associated with adherence, and few studies describe the system‐level variation. Our results underscore the importance of identifying characteristics of health systems and clinics that may contribute to patient adherence to oral therapies.

We observed no statistically significant association between adherence and OS or DFS. Given the lack of survival benefit noted in the trial,^7^ it is not surprising that adherence was not associated with these endpoints. The trial was also not powered to detect differences in survival by adherence.

Patient motivation to participate in a clinical trial may positively impact adherence, and therefore, our findings may not reflect patient adherence to oral therapy in routine, clinical practice settings. For example, in breast cancer trials, non‐adherence to oral endocrine therapies ranges from 8% to 28%, but in clinical practice, more than half of women are non‐adherent to therapy.^8^ There may also be differences in adherence to oral regimens in the adjuvant compared to metastatic setting. Specifically, patients receiving adjuvant therapy––and who have previously received curative surgery––may perceive adherence as having only a modest benefit, and patients with metastatic disease may perceive adherence as more acutely impacting their survival.^27^ To the best of our knowledge, there are few studies of adherence to sorafenib or sunitinib in clinical practice,^29^ nor of other oral therapies used to treat renal cell carcinoma. Finally, we used patient‐reported data to measure adherence, and patients may have completed pill diaries with varying degrees of accuracy. Using microelectronic monitoring systems (i.e., pill bottle with computer cap reader), adherence companion studies of clinical trials^9^, ^10^ report similar estimates of adherence to what we observed. Continuing to refine and evaluate adherence measures in efficacy settings will improve dissemination into practice, mitigating concerns of generalizability to non‐trial populations.

In summary, oral therapies present new challenges to cancer care delivery. Administering cancer therapy has largely shifted from an in‐clinic process monitored by providers to patients’ homes, where providers have no direct supervisory role. Our post hoc analysis of data from a phase III trial for non‐metastatic renal cell carcinoma established an important benchmark measure of adherence in this setting and identified areas for future research. Our findings also highlight several challenges to address in clinical practice as oral therapies become increasingly common. Ongoing efforts to develop and integrate novel tools that monitor oral therapies into clinical workflows will promote safe and effective use.

## DISCLOSURES

Caitlin C. Murphy: Conuslting or advisory role: Freenome. Naomi B. Haas: Consulting or advisory role: Pfizer, Merck Sharp & Dohme; Expert testimony: Eli Lilly (immediate family member). David E. Gerber: Stock and other ownership interests: Gilead Sciences, Consulting or advisory role: Samsung Bioepis, Catalyst Pharmaceutical; Research funding: Astra‐Zeneca, BerGenBio, and Karyopharm. Isaac Alex Bowman: Consulting or advisory role: Foundation Medicine, Inc., Dendreon. Janice P. Dutcher: Consulting or advisory role: Eisai, Merck, Nektar, Amgen, Bristol Myers Squibb, Iovance, Clinigen. No other authors have financial disclosures or potential conflict of interest to report.

## AUTHOR CONTRIBUTIONS

Study conception and design: CCM and DEG; Acquisition of data: MP, NBH, JPD, and RD; Analysis and interpretation of data: All authors; Statistical analysis: HF and CCM; Drafting of manuscript: CCM; Critical revision: All authors.

## Data Availability

The data sets generated, analyzed, and reported in the present paper are available in the NCTN/NCORP Data Archive (https://nctn‐data‐archive.nci.nih.gov).
